# Novel predictor of the occurrence of DKA in T1DM patients without infection: A combination of neutrophil/lymphocyte ratio and white blood cells

**DOI:** 10.1515/biol-2021-0141

**Published:** 2021-12-31

**Authors:** Yiping Cheng, Wenhao Yu, Yuping Zhou, Tao Zhang, Haiyan Chi, Chao Xu

**Affiliations:** Department of Endocrinology and Metabolism, Shandong Provincial Hospital, Cheeloo College of Medicine, Shandong University, 324, Jing 5 Road, Jinan 250021, Shandong, China; Department of Endocrinology and Metabolism, Shandong Provincial Hospital Affiliated to Shandong First Medical University, 324, Jing 5 Road, Jinan 250021, Shandong, China; Department of Endocrinology and Metabolism, Institute of Endocrinology, Shandong Academy of Clinical Medicine, Jinan 250021, Shandong, China; Department of Endocrinology and Metabolism, Shandong Clinical Medical Center of Endocrinology and Metabolism, Jinan 250021, Shandong, China; Department of Biostatistics, School of Public Health, Shandong University, 44 Wenhuaxi Road, Jinan 250012, China; Department of Endocrinology and Metabolism, Weihai Municipal Hospital, 70, Heping Road, Weihai, 264299, China

**Keywords:** lymphocytes, white blood cells, diabetic ketoacidosis

## Abstract

The role of inflammation has been identified in the pathogenesis of diabetic ketoacidosis (DKA). The neutrophil/lymphocyte ratio (NLR) and white blood cells (WBC) can be used to predict a systemic inflammatory response. Changes in NLR and WBC levels have never been explored in type 1 diabetes mellitus (T1DM) patients with DKA and an uninfected state. This retrospective study included a total of 644 participants. NLR and WBC were measured in the control group (*n* = 316) and in T1DM patients with mild-DKA (*n* = 92), severe-DKA (*n* = 52), and non-DKA (*n* = 184) in an uninfected state. Then, we assessed the independent predictors of DKA occurrence in T1DM patients in an uninfected state. The diagnostic performance of variables was determined by receiver operating characteristic curve analysis. Serum NLR of T1DM patients is significantly higher than that of normal controls, and if DKA occurs, NLR increases further and increases with the severity of DKA. In addition to diastolic blood pressure, blood urea nitrogen, glycated hemoglobin (HbA1c), and WBC, NLR was also independently associated with DKA in T1DM patients with an uninfected state (OR = 1.386, 95% CI: 1.127–1.705, *p* = 0.002). Furthermore, the diagnosis analysis showed that except for NLR and WBC, the area under the curve (AUC) of indicators with a statistical difference in patients with and without DKA were 0.747 for DKA diagnosis, and after the addition of NLR and WBC, the AUC was 0.806. The increased NLR level represents a low-cost and highly accessible predictor for DKA in T1DM patients with an uninfected state. The addition of inflammation indicators can play a statistically significant role in the prediction model of the DKA occurrence.

## 1 Introduction

Diabetic ketoacidosis (DKA) is an acute and severe complication of diabetes, and DKA is more likely to occur in type 1 diabetes mellitus (T1DM). Therefore, early detection and treatment of DKA are beneficial to the prognosis of diabetic patients [1,2]. If the simple and low-cost indicators for predicting the occurrence of DKA can be found from routine clinical examination data, the early diagnosis rate of DKA may be improved and its progress can be blocked, thus greatly reducing the harm of DKA.

The most common cause of DKA is infection. In addition, some patients with T1DM may develop DKA and other acute diabetic complications due to irregular use of insulin or the induction of diseases such as infection [3,4]. These patients may also have systemic oxidative stress or inflammatory response, which exacerbates the disorder of the patient’s internal environment [5]. However, DKA in T1DM patients without infection is an uninfected form of systemic inflammatory response syndrome with significantly increased proinflammatory factors [6]. The release of inflammatory factors will weaken the function of pancreatic β cells, while the deficiency of insulin in the DKA state can promote the obvious increase of inflammatory factors by increasing the acute-phase response, thereby forming a vicious circle [7,8]. Therefore, the identification of the exact relationship between inflammatory response and DKA is significant for the early treatment and later recovery of DKA patients.

The neutrophil/lymphocyte ratio (NLR) and white blood cells (WBC) can be used to predict a systemic inflammatory response. The NLR, a new marker of the systemic inflammatory response, integrates the information of neutrophils and lymphocytes. Furthermore, it is easy to obtain NLR and is less affected by physiological factors and specimen treatment [9]. Recent studies have found that NLR is closely related to the occurrence, severity, and prognoses of many acute diseases, such as acute coronary syndrome [10], acute ischemic stroke (AIS) [11], acute pancreatitis [12], acute respiratory distress syndrome [13], acute appendicitis [14], and acute type A aortic dissection [15]. During the development of systemic inflammatory response, WBCs are the effector cells of inflammatory response and the effector mediator of inflammatory conduction. In addition, WBC can regulate the body’s immune function by regulating the cellular immune function and play a key role in the systemic inflammatory response caused by infection or noninfectious factors [16,17,18].

Despite our knowledge about increased inflammation in the context of DKA, the data about the relationship between DKA and NLR, a simple and economical index, are lacking in this population. Changes in NLR and WBC levels have never been explored in T1DM patients with DKA and an uninfected state. Therefore, the aim of this study was to depict the difference in NLR and WBC levels in T1DM patients with DKA and non-DKA in an uninfected state and determine the DKA diagnostic performance of NLR and WBC.

## 2 Subjects and methods

### 2.1 Study subjects

This retrospective study included a total of 644 participants. We first selected 328 T1DM patients hospitalized in Shandong Provincial Hospital affiliated to Shandong University (Jinan, China) and Weihai Municipal Hospital (Weihai, China) from January 2008 to January 2020. With age, sex, and examination time-matched for T1DM participants, we then collected 316 healthy controls from our database of the Health Examination Center. According to the onset characteristics, fasting blood glucose, islet autoantibodies, serum C-peptide, ketone bodies, and blood gas analysis results [19], the individuals were divided into the normal control group (*n* = 316) and T1DM patients with mild-DKA (*n* = 92), severe-DKA (*n* = 52) and non-DKA (*n* = 184) groups. The normal control inclusion criteria were as follows: a healthy person with no physiological, physical, or psychologic abnormalities; Mild-DKA group: T1DM patients with ketosis and an uninfected state; Severe-DKA group: T1DM patients with ketoacidosis and an uninfected state; Non-DKA group: T1DM patients in an uninfected state without ketosis or ketoacidosis. Subjects with serious cardiovascular diseases, such as acute myocardial infarction, cachexia, immune disease, hepatic dysfunction, renal dysfunction (creatinine-based eGFR <90 mL/min/1.73 m^2^), infection, inflammation, or treatment with anti-inflammatory drugs, pregnancy, taking aspirin or statins, were excluded from this study.


**Informed consent:** Informed consent from each patient and healthy control was not required due to the retrospective study design and the use of anonymized data.
**Ethical approval:** The research related to human use has been complied with all the relevant national regulations, institutional policies and in accordance with the tenets of the Helsinki Declaration, and has been approved by the Ethics Committee of Shandong Provincial Hospital affiliated to Shandong University.

### 2.2 Measurements

The authors retrieved all data from previous hospital records of patients and healthy controls admitted to Shandong Provincial Hospital affiliated to Shandong University and Weihai Municipal Hospital; physical examination and extraction and analysis of blood were performed by other experts when patients and healthy controls were admitted to the hospital. After fasting overnight, venous blood was collected. An automatic biochemistry analyzer (Beckman Coulter Analyzer AU58 Series, USA) was used to analyze aspartate transaminase (AST), alanine transaminase (ALT), albumin (ALB), blood urea nitrogen (BUN), creatinine (CREA), total cholesterol (TC), triglycerides (TGs), low-density lipoprotein cholesterol (LDL-c), high-density lipoprotein cholesterol (HDL-c), and uric acid (UA). Glycated hemoglobin (HbA1c) was determined using high-performance liquid chromatography (HPLC) with a hemoglobin A1c analyzer (TOSOH Corporation, Japan). WBC and NLR were analyzed on a blood cell analyzer (Sysmex Corporation XN-2100, Japan). NLR = neutrophil count/lymphocyte count.

### 2.3 Statistical analysis

The Kolmogorov–Smirnov test was used to test the normality of all parameters prior to performing parametric tests. Normally distributed continuous data are represented as the mean ± SD, while nonnormally distributed continuous parameters are represented as the medians with interquartile ranges. Categorical variables are expressed as percentages. To analyze differences between the four groups, data with a normal distribution were tested by one-way ANOVA, and skewed variables were tested by the Kruskal–Wallis H-test. Categorical variables were tested by the Chi-square test. Pearson, Spearman, and partial correlation analyses were used to determine factors that affect NLR and WBC levels. Then, the association between NLR or WBC levels and the occurrence of DKA in T1DM patients were assessed by logistic regression analysis of factors with *p* < 0.05 in single-factor analysis. Notably, we performed collinearity diagnostics of the variables included in the logistic regression analysis. To address the problem of multicollinearity in the multiple linear regression analysis, we used a stepwise model to exclude variables with a minimal explanation of the dependent variable from the combination of multiple collinearities of independent variables. The independent variables included in the logistic regression model are not significantly collinearity. The diagnostic performance of the variables was determined by receiver operating characteristic (ROC) curve analysis. All *p* values were two-tailed, and *p* values less than 0.05 were considered statistically significant. SPSS version 24.0 (SPSS, Chicago, IL, USA) was used for all parametric tests.

## 3 Results

### 3.1 General characteristics of the study population with 644 subjects

**Figure 1:**
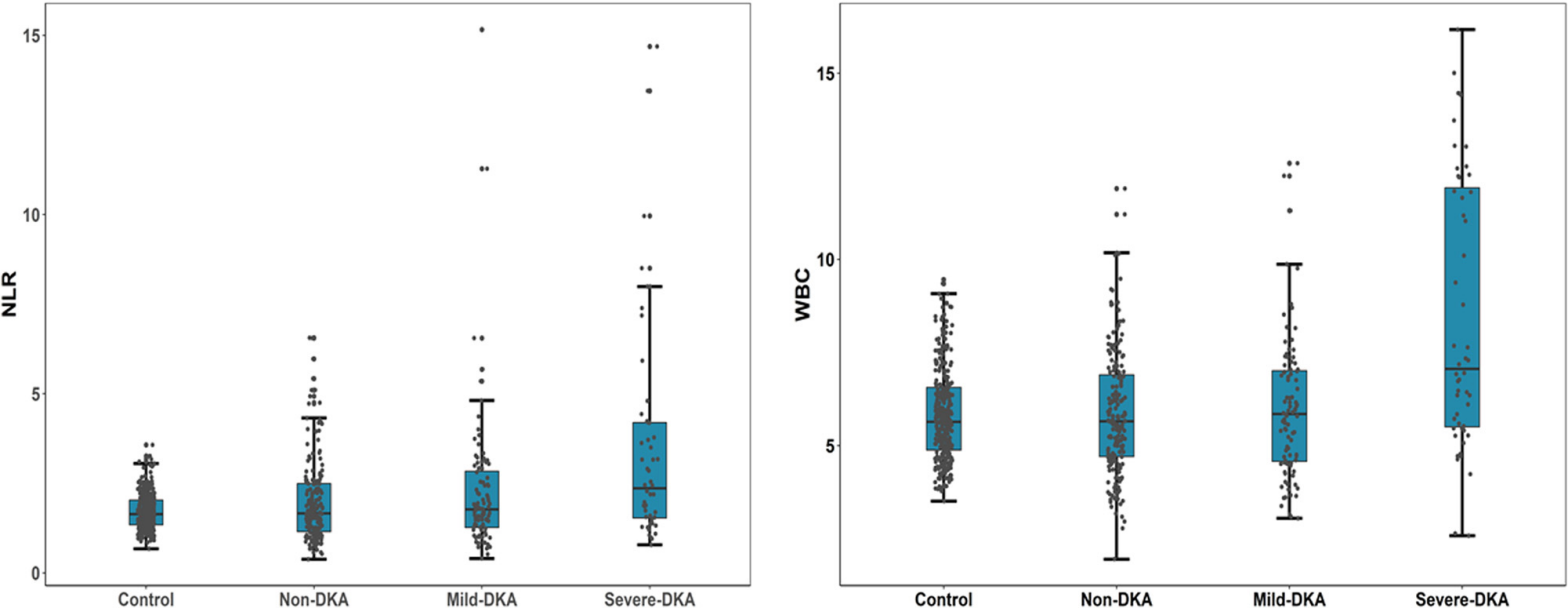
Differences in NLR and WBC levels among the four groups. Serum NLR and WBC levels of T1DM patients are significantly higher than that of normal controls and if DKA occurs, the NLR and WBC levels increase further and increase with the severity of DKA. Abbreviation: NLR, neutrophil/lymphocyte ratio; WBC, white blood cells; Non-DKA group: T1DM patients in an uninfected state without ketosis or ketoacidosis. Mild-DKA group: T1DM patients with ketosis and in an uninfected state. Severe-DKA group: T1DM patients with ketoacidosis and in an uninfected state.

We first analyzed the differences in clinical indicators of the normal control group (*n* = 316) and T1DM patients in an uninfected state with non-DKA (*n* = 184), mild-DKA (*n* = 92), and severe-DKA (*n* = 52) groups (Table 1). Non-DKA patients are older than the normal control group. Compared with T1DM patients without DKA, the patients with DKA were younger, and the patients with ketoacidosis were younger than those with ketosis. Patients with DKA have a shorter duration of diabetes than those without DKA. Compared with the non-DKA group, the SBP and DBP of the mild-DKA group decreased. The ALB level of T1DM patients decreased compared with the control group. Among T1DM patients, the ALB level of patients with DKA was lower than those of patients without DKA. The BUN level of patients with ketosis was lower than those of the control group and patients without DKA, and the BUN level of patients with DKA was higher than those of patients with ketosis. Although the BUN level of patients with DKA was also higher than those of the control group and patients without DKA, there was no statistical difference. Compared with the control group, the CREA level of patients with non-DKA and ketosis decreased, while the CREA level of DKA patients was higher than those of patients with non-DKA and ketosis. The level of HbA1c in DKA patients was higher than those of patients without DKA. Compared with the control group, the TC level of patients with non-DKA and ketoacidosis increased, the TGs level of patients with non-DKA decreased, and the LDL-c or HDL-c level of patients with non-DKA increased. The level of UA in mild-DKA patients was lower than those of the control group and patients without DKA, and the level of UA in severe-DKA patients was higher than those of the other three groups. The WBC and NLR levels of severe-DKA patients were higher than those of patients with non-DKA and ketosis (Figure 1). Next, we divided the diabetic patients into two groups based on whether DKA occurred (Table A1). Regarding the difference in NLR and WBC between the two groups, T1DM patients with DKA had significantly higher serum NLR and WBC levels than those without DKA (1.94 (IQR: 1.35–3.21) vs 1.67 (IQR: 1.15–2.50), *p* = 0.004; 6.23 (IQR: 4.89–7.83) vs 5.66 (IQR: 4.71–6.93), *p* = 0.002, respectively).

**Table 1:** Basic characteristics of the control group and T1DM group

Parameters	Control (*n* = 316)	Non-DKA (*n* = 184)	Mild-DKA (*n* = 92)	Severe-DKA (*n* = 52)	*p*
Age (years)	31.90 ± 14.45	35.17 ± 15.91^a^	31.02 ± 14.70^b^	28.71 ± 13.43^b^	**0.014**
Males% (*n*)	47.15 (149)	50.54 (93)	44.57 (41)	44.23 (23)	0.744
Duration of diabetes (years)	—	5.00 (IQR: 1.00–12.00)	1.00 (IQR: 0.17–6.00)^b^	2.50 (IQR: 0.17–7.00)^b^	<**0.001**
SBP (mmHg)	123.27 ± 10.82	126.24 ± 21.34	120.20 ± 20.05^b^	122.13 ± 19.65	**0.029**
DBP (mmHg)	81.13 ± 8.96	81.21 ± 14.23	75.66 ± 15.18^a,b^	78.38 ± 12.73	**0.001**
AST (U/L)	19.0 (IQR: 16.0–23.0)	19.0 (IQR: 15.0–24.0)	19.0 (IQR: 15.0–24.0)	17.5 (IQR: 15.0–25.75)	0.685
ALT (U/L)	17.0 (IQR: 12.0–25.0)	16 (IQR: 12.0–23.0)	14.0 (IQR: 11.0–23.0)	16.0 (IQR: 13.0–20.5)	0.259
ALB (g/L)	43.30 (IQR: 41.50–45.60)	41.90 (IQR: 38.88–44.03)^a^	39.60 (IQR: 35.88–42.23)^a,b^	36.30 (IQR: 33.55–41.15)^a,b^	<**0.001**
BUN (mmol/L)	4.90 (IQR: 4.13–5.98)	5.00 (IQR: 4.00–6.30)	4.31 (IQR: 2.70–5.41)^a,b^	5.55 (IQR: 3.74–7.04)^c^	<**0.001**
CREA (µmol/L)	68.00 (IQR: 59.38–76.96)	61.00 (IQR: 48.89–73.50)^a^	55.90 (IQR: 47.30–68.00)^a^	69.10 (IQR: 58.23–96.93)^b,c^	<**0.001**
HbA1c (%)	—	9.10 (IQR: 7.68–11.43)	11.70 (IQR: 9.60–13.10)^b^	13.20 (IQR: 10.75–15.00)^b^	<**0.001**
TC (mmol/L)	4.64 ± 0.76	4.97 ± 1.76^a^	4.68 ± 1.45	5.13 ± 2.14^a^	**0.017**
TGs (mmol/L)	1.03 (IQR: 0.82–1.36)	0.87 (IQR: 0.64–1.30)^a^	0.88 (IQR: 0.65–1.37)	1.07 (IQR: 0.85–1.83)	**0.008**
HDL-c (mmol/L)	1.22 ± 0.25	1.42 ± 0.36^a^	1.28 ± 0.40^b^	1.34 ± 0.87	<**0.001**
LDL-c (mmol/L)	2.54 ± 0.56	2.84 ± 1.33^a^	2.75 ± 1.07	2.82 ± 1.01	**0.004**
UA (mmol/L)	269.52 ± 62.27	260.98 ± 81.38	212.61 ± 76.96^a,b^	345.89 ± 173.09^a,b,c^	<**0.001**

One-way ANOVA, Kruskal–Wallis H-test and Chi-square test. Mean ± standard deviation or median (IQR: interquartile range) and *n* (%). Non-DKA group: T1DM patients in an uninfected state without ketosis or ketoacidosis. Mild-DKA group: T1DM patients with ketosis and in an uninfected state. Severe-DKA group: T1DM patients with ketoacidosis and in an uninfected state. SBP, systolic blood pressure; DBP, diastolic blood pressure; AST, aspartate transaminase; ALT, alanine transaminase; ALB, albumin; BUN, blood urea nitrogen; CREA, creatinine; HbA1c, glycated hemoglobin; TC, total cholesterol; TGs, triglycerides; HDL-c, high-density lipoprotein cholesterol; LDL-c, low-density lipoprotein cholesterol; UA, uric acid. Differences with a probability value of *p* < 0.05 were considered statistically significant and are given in bold. Compared with the control group, ^a^ *p* < 0.05. Compared with the Non-DKA group, ^b^ *p* < 0.05. Compared with the Mild-DKA group, ^c^ *p* < 0.05.

In addition, there were no differences in gender, AST, or ALT levels between the four groups.

Our findings indicated that serum NLR and the WBC level of T1DM patients is significantly higher than that of normal controls, and if DKA occurs, the NLR and the WBC level increase further and increase with the severity of DKA.

### 3.2 Correlation between NLR or WBC and the clinical variables

The correlations between NLR and clinical parameters in patients with T1DM are shown in Table 2. According to these results, serum NLR was positively correlated with age, duration of diabetes, SBP, BUN, CREA, TC, TGs, LDL-c, UA, and WBC and negatively correlated with AST, ALT, and ALB. As previously reported in patients with type 2 diabetes mellitus [20], Sefil et al. showed that the duration of diabetes was related to NLR levels. In addition, age and duration of diabetes had a significant correlation in T1DM patients (*r* = 0.367; *p* <0.001). To avoid the influence of age and duration of diabetes on NLR levels and eliminate potential confounding factors, we adjusted for age and duration of diabetes. After adjustment, CREA, HbA1c, and WBC positively correlated between NLR (*r* = 0.184, *p* = 0.003; *r* = 0.125, *p* = 0.043; *r* = 0.554, *p* <0.001, respectively) and ALB negatively correlated with NLR (*r* = −0.179, *p* = 0.004), whereas the other positive or negative correlations above were no longer significant.

**Table 2:** Correlation of serum NLR levels with the clinical variables

Parameters	NLR		Adjusted NLR	
	*r*	*p*	*r*	*p*
Age (years)	0.114	**0.038**	—	—
Duration of diabetes (years)	0.217	<**0.001**	—	—
Sex	−0.027	0.626	0.072	0.246
SBP (mmHg)	0.218	<**0.001**	0.086	0.164
DBP (mmHg)	0.150	0.007	0.060	0.330
AST (U/L)	−0.131	**0.019**	−0.046	0.452
ALT (U/L)	−0.119	**0.033**	−0.052	0.403
ALB (g/L)	−0.127	**0.022**	−0.179	**0.004**
BUN (mmol/L)	0.202	<**0.001**	0.086	0.161
CREA (µmol/L)	0.257	<**0.001**	0.184	**0.003**
HbA1c (%)	0.031	0.584	0.125	**0.043**
TC (mmol/L)	0.176	**0.002**	0.059	0.336
TGs (mmol/L)	0.198	**0.001**	0.097	0.118
HDL-c (mmol/L)	−0.038	0.513	−0.080	0.196
LDL-c (mmol/L)	0.174	**0.003**	0.065	0.295
UA (mmol/L)	0.181	**0.001**	0.054	0.384
WBC (10^9^/L)	0.531	<**0.001**	0.554	<**0.001**

SBP, Systolic blood pressure; DBP, diastolic blood pressure; AST, aspartate transaminase; ALT, alanine transaminase; ALB, albumin; BUN, blood urea nitrogen; CREA, creatinine; HbA1c, glycated hemoglobin; TC, total cholesterol; TGs, triglycerides; HDL-c, high-density lipoprotein cholesterol; LDL-c, low-density lipoprotein cholesterol; UA, uric acid; NLR, neutrophil/lymphocyte ratio; WBC, white blood cells. Differences with a probability value of *p* < 0.05 were considered statistically significant and are given in bold.

Considering the strong correlation between WBC and NLR, we assessed factors associated with WBC and found that WBC was positively correlated with NLR, HbA1c, TC, TGs, LDL-c, and UA and negatively correlated with ALB after adjustment for age and duration of diabetes (Table 3).

**Table 3:** Correlation of serum WBC levels with the clinical variables

Parameters	WBC		Adjusted WBC	
	*r*	*p*	*r*	*p*
Age (years)	0.006	0.919	—	—
Duration of diabetes (years)	0.177	**0.001**	—	—
Sex	−0.010	0.861	0.011	0.860
SBP (mmHg)	0.150	**0.007**	0.022	0.718
DBP (mmHg)	0.157	**0.004**	0.070	0.259
AST (U/L)	−0.130	**0.020**	−0.058	0.345
ALT (U/L)	−0.134	**0.016**	−0.065	0.294
ALB (g/L)	−0.155	**0.005**	−0.139	**0.024**
BUN (mmol/L)	0.195	<**0.001**	0.114	0.064
CREA (µmol/L)	0.267	<**0.001**	0.116	0.059
HbA1c (%)	0.093	0.102	0.154	**0.012**
TC (mmol/L)	0.209	<**0.001**	0.134	**0.029**
TGs (mmol/L)	0.285	<**0.001**	0.203	**0.001**
HDL-c (mmol/L)	−0.058	0.321	−0.047	0.447
LDL-c (mmol/L)	0.208	<**0.001**	0.126	**0.040**
UA (mmol/L)	0.253	<**0.001**	0.253	<**0.001**
NLR	0.531	<**0.001**	0.554	<**0.001**

SBP, systolic blood pressure; DBP, diastolic blood pressure; AST, aspartate transaminase; ALT, alanine transaminase; ALB, albumin; BUN, blood urea nitrogen; CREA, creatinine; HbA1c, glycated hemoglobin; TC, total cholesterol; TGs, triglycerides; HDL-c, high-density lipoprotein cholesterol; LDL-c, low-density lipoprotein cholesterol; UA, uric acid; NLR, neutrophil/lymphocyte ratio; WBC, white blood cells. Differences with a probability value of *p* < 0.05 were considered statistically significant and are given in bold.

### 3.3 Correlation between serum NLR and WBC levels and the occurrence of diabetic ketoacidosis in T1DM patients in an uninfected state

Similarly, due to the strong correlation between WBC and NLR, we included WBC and NLR into the multiple logistic regression model, respectively. Table 4 demonstrates the results of the analysis carried out to identify the correlation between serum NLR levels and the occurrence of DKA in T1DM patients. The independent variables entering the equation included age, duration of diabetes, SBP, DBP, ALB, BUN, HbA1c, HDL-c, and NLR. Consequently, HbA1c and NLR were independent risk factors for the occurrence of DKA in T1DM patients, while DBP and BUN were protective factors. Table 5 demonstrates the results of the analysis carried out to identify the correlation between serum WBC levels and the occurrence of DKA in T1DM patients. The independent variables entering the equation included age, duration of diabetes, SBP, DBP, ALB, BUN, HbA1c, HDL-c, and WBC. HbA1c and WBC were independent risk factors for the occurrence of DKA in T1DM patients, while DBP and BUN were protective factors.

**Table 4:** Predictors of DKA in T1DM patients in multivariate logistic regression analysis with DKA as a dependent variable; and age, duration of diabetes, SBP, DBP, ALB, BUN, HbA1c, HDL-c, and NLR as independent variables

Parameters	OR	*p*	95% CI
Age (years)	0.994	0.590	0.973–1.016
Duration of diabetes (years)	0.957	0.115	0.906–1.011
SBP (mmHg)	1.011	0.339	0.989–1.034
DBP (mmHg)	0.968	**0.026**	0.940–0.996
ALB (g/L)	0.973	0.278	0.926–1.022
BUN (mmol/L)	0.879	**0.028**	0.783–0.986
HbA1c (%)	1.401	<**0.001**	1.237–1.588
HDL-c (mmol/L)	0.805	0.469	0.447–1.449
NLR	1.386	**0.002**	1.127–1.705

SBP, systolic blood pressure; DBP, diastolic blood pressure; ALB, albumin; BUN, blood urea nitrogen; HbA1c, glycated hemoglobin; HDL-c, high-density lipoprotein cholesterol; NLR, neutrophil/lymphocyte ratio; OR, odds ratio; CI, confidence interval. Differences with a probability value of *p* < 0.05 were considered statistically significant and are given in bold.

**Table 5:** Predictors of DKA in T1DM patients in multivariate logistic regression analysis with DKA as the dependent variable; and age, duration of diabetes, SBP, DBP, ALB, BUN, HbA1c, HDL-c, and WBC as independent variables

Parameters	OR	*p*	95% CI
Age (years)	0.997	0.798	0.975–1.019
Duration of diabetes (years)	0.948	0.055	0.897–1.001
SBP (mmHg)	1.016	0.184	0.993–1.039
DBP (mmHg)	0.963	**0.012**	0.935–0.992
ALB (g/L)	0.969	0.212	0.921–1.018
BUN (mmol/L)	0.855	**0.015**	0.754–0.971
HbA1c (%)	1.406	<**0.001**	1.238–1.597
HDL-c (mmol/L)	0.838	0.543	0.474–1.481
WBC (10^9^/L)	1.337	<**0.001**	1.160–1.540

SBP, systolic blood pressure; DBP, diastolic blood pressure; ALB, albumin; BUN, blood urea nitrogen; HbA1c, glycated hemoglobin; HDL-c, high-density lipoprotein cholesterol; WBC, white blood cell; OR, odds ratio; CI, confidence interval. Differences with a probability value of *p* < 0.05 were considered statistically significant and are given in bold.

### 3.4 ROC curve of the predicted variables associated with the occurrence of DKA in T1DM patients in an uninfected state

**Figure 2:**
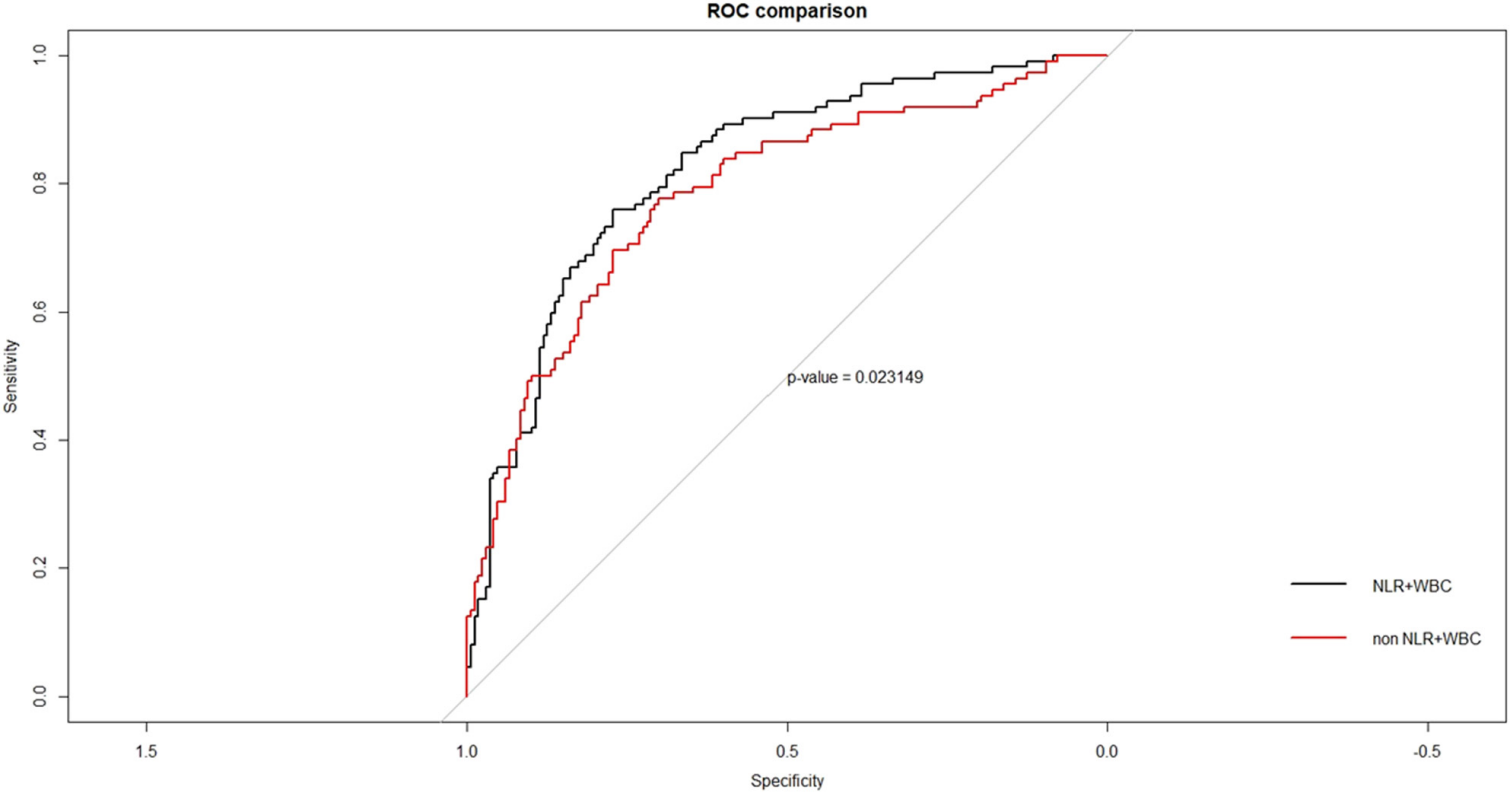
ROC curve of the predicted variables associated with the occurrence of DKA in T1DM patients. The diagnosis analysis showed that except for NLR and WBC, the area under curve (AUC) of indicators with the statistical difference in patients with and without DKA were 0.747 for DKA diagnosis, and after the addition of NLR and WBC, the AUC was 0.806. The addition of inflammation indicators can play a statistically significant gain in the prediction model of the occurrence of DKA. Abbreviation: ROC, receiver operating characteristic curve analysis; NLR, neutrophil/lymphocyte ratio; WBC, white blood cells. Differences with a probability value of *p* < 0.05 were considered statistically significant.

Furthermore, the diagnosis analysis showed that except for NLR and WBC, the area under the curve (AUC) of indicators with the statistical difference in patients with and without DKA were 0.747 for DKA diagnosis, and after the addition of NLR and WBC, the AUC was 0.806 (Figure 2). The addition of inflammation indicators can play a statistically significant gain in the prediction model of DKA occurrence.

## 4 Discussion

The current study investigated NLR and WBC levels in normal people and in T1DM patients with non-DKA, mild-DKA, and severe-DKA in an uninfected state. Intriguingly, we found that serum NLR and the WBC level of T1DM patients is significantly higher than that of normal controls, and if DKA occurs, the NLR and WBC level increase further and increase with the severity of DKA. The increased serum NLR and WBC levels were independently associated with the occurrence of DKA in T1DM patients in an uninfected state, and the addition of NLR and WBC can play a statistically significant role in the prediction model of the occurrence of DKA. To the best of our knowledge, we have not seen any report describing the NLR levels in T1DM patients with DKA and an uninfected state.

There is increasing evidence that easily obtainable laboratory biomarkers can have a predictive role and be used for diagnosis and outcome prediction in various medical conditions. NLR, a relatively inexpensive marker of inflammation, has recently been explored as a predictor of mortality in patients who undergo percutaneous coronary intervention and can aid in the risk stratification and prognosis of patients diagnosed with acute coronary syndromes [21]. Moreover, NLR is an independent predictor of contrast-induced acute nephropathy in patients with acute myocardial infarction [22]. Remarkably, Lattanzi found that higher NLR levels upon admission were independently associated with poor outcomes at 30 days after the acute intracerebral hemorrhage onset [23]. The inflammatory response that occurs after cerebral infarct plays a critical role in stroke pathobiology and can affect the outcome of the recanalization procedure [24]. Furthermore, NLR showed good discriminatory power in predicting symptomatic hemorrhagic transformation, a life-threatening complication of AIS, in patients with AIS undergoing revascularization [25]. In cancer patients, higher pretreatment NLR levels are associated with poor survival outcomes. A study based on a retrospective cohort of 5,363 patients treated at the Moffitt Cancer Center (Tampa, FL) found that NLR demonstrated stronger associations with survival among African-American patients, patients receiving radiation therapy, stage IV patients, and melanoma patients when compared with the overall study population. In other words, for patients with certain demographic and clinical characteristics, NLR may have a greater prognostic value [26]. A retrospective study evaluated 240 patients with newly diagnosed metastatic colorectal cancer (stage IV), who underwent surgical resection, and found a high SII value calculated based on preoperative laboratory data regarding platelet, neutrophil, and lymphocyte counts that independently predicted poor clinical outcomes among patients with metastatic colorectal cancer [27]. Likewise, NLR has been shown as a simple promising method to predict bacteremia in adult patients admitted to the Emergency Department with suspected community-acquired bacteremia [28].

As is well known, both T1DM and type 2 diabetes mellitus (T2DM) are considered to be systemic inflammatory states [29,30]. Studies have shown that the increased release of inflammatory factors (such as TNF-α, IL-6, C-reactive protein) and the decreased release of anti-inflammatory cytokines (such as IL-10) can lead to the increased activation of adipose tissue, increased insulin resistance, and further progression to diabetes [31,32]. In our study, T1DM patients with an uninfected state had significantly higher serum NLR levels than individuals in the normal control group (1.80 [IQR: 1.24–2.67] vs 1.64 [IQR: 1.34–2.03], *p* <0.001). In addition to the explanation that T1DM is a chronic inflammatory disease and NLR reflects the state of systemic inflammation, we suspected that the increase of NLR in T1DM patients may also be related to poor blood glucose regulation. Sefil et al. [20]. found that NLR was higher in T2DM patients with HbA1c levels >7% than those ≤7%. Similarly, we found a positive correlation between NLR and HbA1c in T1DM patients after adjustment for age and duration of diabetes. A previous study about the relationship of NLR with aortic stiffness found that the NLR levels were significantly higher in T1DM patients than in healthy individuals, which was consistent with our findings. It was reported that the increased blood glucose caused a drop in lymphocyte levels [33], and lymphocytes did not proliferate enough in diabetic patients [34]. In the context of diabetes, inflammatory responses can be intensified by the hyperglycemic state [31]. Nonetheless, the exact mechanism of the increased NLR in T1DM patients in an uninfected state still needs further investigation, as our conjectures cannot completely elucidate this point.

Furthermore, T1DM patients with DKA and an uninfected state had significantly higher serum NLR and WBC levels than those in the non-DKA group, and NLR and WBC levels increased with the severity of DKA. In addition, the increased serum NLR and WBC levels were independent predictors of the occurrence of DKA in T1DM patients with an uninfected state, and the addition of inflammation indicators can play a statistically significant gain in the prediction model of the occurrence of DKA. We have a few possible explanations for these findings. First, neutrophils are involved in the body’s inflammatory response, while lymphocytes as the inflammatory regulator can protect endothelial cells and reduce the inflammatory response. The ratio of neutrophils and lymphocytes reflects the balance between the inflammatory activator and the inflammatory regulator. The higher the NLR, the more severe the inflammatory response [35]. The uninfected state of DKA can be identified as a serious form of systemic inflammatory response syndrome with significantly increased proinflammatory factors [6]. Therefore, our study suggests that the NLR may be an indicator of the underlying severity of acute systemic inflammation in DKA patients with an uninfected state. Second, elaborated cytokines can trigger inflammatory responses during hypoxemic episodes [36]. We suspect that DKA leads to an abnormality in the oxygen system and the occurrence of inflammation. In addition, hypoxia may inhibit the apoptosis of neutrophils in DKA patients, as in the asthma attack [37]. Third, the number of neutrophils and lymphocytes is regulated by the autonomic nervous system. There are adrenergic receptors on the surface of neutrophils, and the neutrophilic number and function are regulated by sympathetic nerves, while there are cholinergic receptors on the surface of lymphocytes that are regulated by parasympathetic nerves [38]. Increased sympathetic nerve activity can produce more neutrophils and proinflammatory substances [39,40]. When DKA occurs, the sympathetic nerve is excited, which stimulates the proliferation of neutrophils in the bone marrow. Fourth, the acute hyperglycemia and blood glucose fluctuations promote the increase of reactive oxygen species (ROS) in the state of DKA [41]. ROS can damage the DNA of peripheral blood lymphocytes, lead to the apoptosis of lymphocytes, and affect their proliferation [42,43]. Fifth, WBC can regulate the body’s immune function by regulating the cellular immune function and play a key role in the systemic inflammatory response caused by infection or noninfectious factors [16,17,18], which reflects the severity of the inflammatory response.

The death of DKA patients due to delayed diagnosis and lack of reasonable treatment is still common. Therefore, analyzing the risk factors for DKA in T1DM patients with an uninfected state is of great significance for early prevention, early diagnosis, and treatment. In addition to NLR, WBC and HbA1c were also independent predictors of DKA occurrence in T1DM patients in an uninfected state. Poor blood glucose control indicates severe metabolic disorders and severe insulin deficiency in the body. Consistent with other studies [44,45], a higher frequency of DKA was strongly associated with poorer glycemic control. This strongly suggests strengthening the monitoring frequency and controlling the level of blood glucose in T1DM patients. In addition, DBP and BUN were protective factors of the occurrence of DKA in T1DM patients in an uninfected state. During DKA, high blood glucose and ketones increase the excretion of glucose and ketones in the kidneys. Osmotic diuresis occurs and a large amount of water is lost, leading to a decreased effective circulating blood volume. Peripheral circulatory failure and hypovolemic shock can occur due to blood volume reduction and microcirculatory disturbances caused by acidosis. Lower blood pressure and renal perfusion can lead to acute renal failure [46]. Therefore, prevention and treatment of shock are very important for DKA patients. The relationship between WBC levels and blood glucose control has been studied by several groups [47,48]. When DKA occurs in a body without infection, the mechanism of increased WBC levels may be similar to that of increased neutrophil levels, but the existence of other causes, except for the activation of the immune system, cannot be still denied.

Interestingly, we found that serum NLR was positively correlated with SBP and DBP in T1DM patients with an uninfected state before adjusting for age and duration of diabetes (Table 2). The relationship between NLR and hypertension has been thoroughly investigated, and NLR is a reliable predictor of hypertension such as resistant hypertension [49,50]. The probable cause is that neutrophils secrete mediators of the inflammatory response, such as elastase, which are associated with plaque rupture and may contribute to an increased risk of hypertension [51,52]. In addition, neutrophils can release ROS that causes oxidative stress, which is an important factor in the pathogenesis of hypertension [53,54]. Additionally, UA is the final product of human purine catabolism and UA crystals deposited in tissues are the strong inflammatory stimulant. Elevated levels of UA show a significant relationship with the increase of certain inflammation markers and oxidative stress indicators [55,56]. In the form of monosodium urate crystals, UA can promote neutrophil recruitment through the complement pathway [57]. Therefore, our findings that UA was significantly positively correlated with serum NLR levels in T1DM patients in an uninfected state may be explained by the above mechanism. When DKA occurs in T1DM patients, the body suffers from endogenous insulin deficiency, glucose metabolism disorder, and increased adipose tissue mobilization. As mentioned earlier, inflammation plays an important role in the pathogenesis of DKA. Studies have found that some patients with severe systemic inflammatory response syndrome may have decreased serum albumin levels, and the degree of change is related to the prognosis of the disease [58]. This study shows that the ALB level of T1DM patients decreased compared with the control group, the ALB level of patients with DKA was lower than that of patients without DKA, and ALB was negatively correlated with NLR. The relationship between NLR and ALB levels needs to be explored in depth. Interestingly, our study showed a positive correlation between NLR and TC, TGs, LDL-c; so, whether the inflammatory response affects blood lipid levels in DKA patients? This may be a fascinating research direction.

It is incontestable that this study has many limitations. First, only one measurement of neutrophil and lymphocyte count and one calculation of NLR were carried out in the analysis. There was no monitoring of the dynamic trend of NLR. Second, selection bias may arise because our data are only from one hospital. Additionally, the sample size was small, and we look forward to future verification in large samples and multicenter studies. Third, our data came from a retrospective study. To fully understand the role of NLR in T1DM patients with DKA and an uninfected state, basic large-scale prospective studies should be conducted.

## 5 Conclusion

In summary, our study demonstrates that NLR and WBC levels increased in T1DM patients with DKA and an uninfected state and increased with DKA severity. NLR and WBC levels can be used as a simple and economical predictor for DKA occurrence in T1DM patients in an uninfected state. When DKA occurs, there may be a severe inflammatory reaction. In addition to lowering blood glucose, anti-inflammatory drugs are expected to relieve the internal environment disorder to a certain extent.

## Appendix

**Table A1:** Basic characteristics of the DKA group and non-DKA group

	DKA (*n* = 144)	Non-DKA (*n* = 184)	*p*
Age (years)	30.19 ± 14.25	35.17 ± 15.91	**0.003**
Males% (*n*)	44.44 (64)	50.54 (93)	0.272
Duration of diabetes (years)	1.25 (IQR: 0.17–6.75)	5.00 (IQR: 1.00–12.00)	<**0.001**
SBP (mmHg)	120.90 ± 19.86	126.24 ± 21.34	**0.021**
DBP (mmHg)	76.65 ± 14.36	81.21 ± 14.23	**0.004**
AST (U/L)	18.0 (IQR:15.0–25.00)	19.0 (IQR:15.0–24.0)	0.466
ALT (U/L)	15.0 (IQR:11.0–21.0)	16.0 (IQR:12.0–23.0)	0.177
ALB (g/L)	38.20 (IQR:35.30–42.00)	41.90 (IQR:38.88–44.00)	<**0.001**
BUN (mmol/L)	4.63 (IQR:3.20–6.10)	5.00 (IQR:4.00–6.30)	**0.013**
CREA (µmol/L)	62.00 (IQR:49.57–73.00)	61.00 (IQR:48.89–73.50)	0.983
HbA1c (%)	12.25 (IQR:10.10–13.83)	9.10 (IQR:7.68–11.43)	<**0.001**
TC (mmol/L)	4.83 ± 1.72	4.97 ± 1.76	0.514
TGs (mmol/L)	0.94 (IQR:0.68–1.46)	0.87 (IQR:0.64–1.30)	0.144
HDL-c (mmol/L)	1.30 ± 0.60	1.42 ± 0.36	**0.035**
LDL-c (mmol/L)	2.77 ± 1.05	2.84 ± 1.33	0.647
UA (mmol/L)	259.41 ± 135.19	260.98 ± 81.38	0.899
NLR	1.90 (IQR:1.34–3.16)	1.66 (IQR:1.14–2.49)	**0.011**
WBC (10^9^/L)	6.14 (IQR:4.80–7.59)	5.66 (IQR:4.70–6.95)	**0.010**

SBP, Systolic blood pressure; DBP, diastolic blood pressure; AST, aspartate transaminase; ALT, alanine transaminase; ALB, albumin; BUN, blood urea nitrogen; CREA, creatinine; HbA1c, glycated hemoglobin; TC, total cholesterol; TGs, triglycerides; HDL-c, high-density lipoprotein cholesterol; LDL-c, low-density lipoprotein cholesterol; UA, uric acid; NLR, neutrophil/lymphocyte ratio; WBC, white blood cells. Differences with a probability value of *p* < 0.05 were considered statistically significant and are given in bold.
